# ConvLoRa: Convolutional Neural Network-Based Collision Demodulation for LoRa Uplinks in LEO-IoT

**DOI:** 10.3390/s26061919

**Published:** 2026-03-18

**Authors:** Tao Hong, Linkun Xu, Xiaodi Yu, Jiawei Shen, Gengxin Zhang

**Affiliations:** School of Communication and Information Engineering, Nanjing University of Posts and Telecommunications, Nanjing 210049, China

**Keywords:** LEO, Sat-IoT, LoRa, signal separation, machine learning, CNN

## Abstract

Satellites supporting IoT connectivity may need to serve a large population of LoRa terminals, where collisions among packets using the same spreading factor (SF) can severely degrade uplink reliability. The ALOHA-based access used in LEO-IoT leads to frequent collisions under massive terminal access, which limits system capacity. Conventional signal separation methods that rely on the capture effect typically require a sufficiently large power difference between colliding signals. However, due to the channel characteristics of LEO links, this condition is often difficult to satisfy. We propose ConvLoRa, a collision demodulation method for co-SF LoRa uplink signals in LEO-IoT based on a fully convolutional neural network (FCN). To improve robustness to synchronization deviations, ConvLoRa uses an up-chirp in the preamble as a reference for feature matching, and employs data augmentation to emulate synchronization deviations during training. In addition, a multi-task design is adopted to estimate the payload length with minimal introduction of extra network parameters. Experiments show that ConvLoRa achieves lower demodulation bit error rate (BER) under collision conditions compared with baselines, including CoRa and SIC-based receivers. Under the condition of a two-signal collision with SNR = −9 dB and SF = 8, the BER of the proposed method is 21% that of CoRa and 28% that of the SIC-based method.

## 1. Introduction

The Satellite Internet of Things (Sat-IoT), serving as an alternative or complement to terrestrial IoT systems, has become a major research focus in the field of IoT technologies. Compared with terrestrial IoT, Sat-IoT offers several advantages, such as global coverage and high reliability [1]. Among satellite constellations deployed in geostationary (GEO), medium (MEO), and low Earth orbits (LEO), LEO satellites are particularly attractive for IoT applications. This appeal arises from their intrinsic advantages, such as low latency, high bandwidth, low transmission cost, strong interference resilience, and compatibility with low-power terminals. These strengths make LEO satellites highly promising, especially in scenarios requiring global connectivity and efficient communication. Consequently, Low Earth Orbit IoT (LEO-IoT) is widely regarded as a major trend in the future development of Sat-IoT.

In terrestrial IoT systems, LoRa has attracted considerable research interest. LoRa employs chirp spread spectrum (CSS) modulation, where information symbols are encoded as cyclic frequency shifts of a linearly frequency-modulated chirp signal. Due to its advantages in low power consumption, long communication range, low cost, and strong interference resilience, LoRa is well suited to the application requirements of LEO satellite IoT systems, especially in global, remote, and connectivity-constrained environments. Consequently, the adoption of LoRa in LEO-IoT scenarios has received increasing attention from both academia and industry, resulting in a series of recent research efforts.

Although LoRa demonstrates considerable potential in LEO-IoT applications, the issue of signal collisions becomes increasingly severe as the number of IoT terminals grows. Moreover, because LoRa employs an ALOHA-based random access mechanism, there is neither centralized scheduling nor time-frequency coordination among terminals. When the satellite coverage footprint is large, even if the activity probability of individual nodes is low, the likelihood of simultaneous transmissions across different nodes remains high, leading to frequent collision events. As reported in [2], in LEO scenarios, the access success rate of LoRa terminals degrades exponentially with increasing ground terminal density due to collision. When the density reaches $10^{-5}\;m^{-2}$, the collision probability for LoRa signals with SF=8 (Spreading Factor) exceeds 20%. According to reference [1], the probability of triple collisions in LoRaWAN is considerably smaller than that of double collisions. A similar trend holds for higher-order collisions, i.e., the probability of quadruple collisions is also considerably smaller than that of triple collisions.

In recent years, many studies have investigated the demodulation of LoRa frames under collision conditions in order to improve the overall bandwidth utilization of LoRa networks, thereby enhancing the network capacity of LoRaWAN. However, these methods usually target terrestrial IoT channel conditions, and many existing algorithms are constrained by two primary limitations: the requirement for large power disparities and the need for precise synchronization. Moreover, they often lack discussion of signal detection and synchronization; if the frame-detection algorithm or synchronization algorithm fails to detect interfering frames, these methods will no longer work.

In this paper, we propose ConvLoRa, a neural network-based collision decoding framework designed to accurately classify LoRa symbols using a convolutional neural network (CNN). ConvLoRa is specifically developed for realistic symbols that are subject to same-SF collisions in the uplink of LEO satellite channels. ConvLoRa will be deployed at the satellite side to assist the satellite in performing LoRa signal separation and demodulation more effectively. The proposed method enhances symbol demodulation accuracy in the congested LEO-IoT environment, thereby significantly reducing the bit error rate (BER). Our main contributions are summarized as follows:

- Proposing a CNN model named FFT-CNN tailored for feature extraction;
- Estimating the number of symbols without introducing additional network parameters;
- Enhancing the network’s robustness to synchronization deviations through data augmentation.

The remainder of this paper is organized as follows. Section 2 reviews the existing studies on LoRa signal separation and demodulation, as well as several machine-learning approaches for LoRa signal processing. Section 3 describes the system model in detail, including the channel conditions of LEO-IoT, the LoRa frame structure and waveform characteristics. Section 4 presents the proposed LoRa demodulation scheme and the training process for the neural network. Section 5 provides the evaluation results based on real-world and simulated datasets, followed by a discussion.

## 2. Related Studies

### 2.1. Satellite IoT

Recent years have witnessed increasing research interest in Sat-IoT systems, which aim to provide large-scale connectivity for IoT devices in remote and infrastructure-limited areas. A comprehensive overview of satellite IoT technologies, communication standards, and open research challenges is presented in [3], highlighting the potential of integrating non-terrestrial networks with IoT services to achieve global coverage. Several studies have also explored the practical deployment and application scenarios of satellite-enabled IoT systems. For example, Galli et al. [4] investigated the use of satellite IoT technologies for monitoring and tracking athletes in extreme environments, demonstrating the feasibility of satellite-assisted sensing and data collection in challenging scenarios. Similarly, Giannetti et al. [5] proposed an IoT node design that enables real-time LoRa communication via GEO satellite links, showing the potential of satellite–LoRa integration for remote monitoring applications such as agri-food supply chain tracking. In addition, Zhang et al. [6] studied adaptive resource optimization strategies for LoRa-enabled LEO satellite IoT systems operating in highly dynamic environments, addressing challenges related to resource allocation and network performance under satellite mobility. These studies collectively demonstrate the growing importance of satellite IoT systems and the feasibility of combining LoRa technology with satellite communication to support large-scale IoT connectivity.

### 2.2. Comparison with NB-IoT and LoRa

Similar to LoRa, NB-IoT has also been widely considered for LEO-IoT communications [7]. In NB-IoT, the downlink adopts OFDM modulation, whereas the uplink employs SC-FDMA, while LoRa utilizes Chirp Spread Spectrum (CSS) modulation for both uplink and downlink transmissions. In LEO-IoT scenarios, an NB-IoT terminal first transmits an uplink preamble over the NPRACH to initiate the random access procedure, enabling the estimation of time of arrival (ToA) and carrier frequency offset (CFO), thereby achieving uplink time–frequency synchronization. Based on this random access process, the satellite estimates the Timing Advance (TA) and schedules subsequent NPUSCH transmissions, allowing the terminal to accomplish uplink data access in LEO channels characterized by propagation delay and Doppler effects [8]. In contrast, LoRa uplink access is typically based on an ALOHA-type grant-free scheme. Meanwhile, the preamble and payload are transmitted within the same signal in LoRa, and Doppler compensation in LoRa systems can be implemented in a relatively straightforward manner. From an uplink waveform perspective, the DFT-s-OFDM signal employed in NB-IoT is inherently more sensitive to residual Doppler-induced carrier frequency offsets than CSS-modulated LoRa signals. In LEO-IoT scenarios, the NB-IoT uplink typically relies on GNSS-assisted Doppler pre-compensation, where the UE proactively adjusts the carrier frequency, prior to NPUSCH transmission, according to satellite ephemeris information in order to mitigate frequency shifts induced by high-speed satellite motion. Subsequently, differential Doppler is alleviated through beam partitioning, and frequency domain channel estimation and Doppler tracking are performed using DMRS embedded in NPUSCH, thereby facilitating effective Doppler compensation [9]. In LoRa systems, Doppler shifts are primarily translated into symbol timing drifts after dechirping, which can be handled by low-complexity synchronization and drift-tracking algorithms [10].

Regarding collision resolution in NB-IoT, since the preamble and payload are transmitted separately, collision mitigation mainly focuses on NPRACH preamble separation or on reducing collision probability through time–channel load balancing. Ref. [11] proposed an Adaptive Coverage Enhancement (ACE) scheme that jointly optimizes the maximum preamble repetition number and back-off window size to temporally distribute failed access attempts across different RAOs, thereby balancing the random access load. Preamble collisions are further mitigated by restricting excessive repetitions within a single RAO and introducing randomized back-off, which reduces simultaneous retransmissions and improves RACH success probability. For preamble separation, Lin [12] proposed a symbol-level matched filtering (SLMF)-based detection framework that separates superimposed NPRACH preambles by exploiting local phase coherence and applying Neyman–Pearson hypothesis testing on accumulated matched filter outputs. Reliable preamble separation under multi-user CFO-induced ICI is further achieved by jointly estimating timing and frequency offsets via JMLE, followed by interference regeneration and closed-loop CFO compensation. Due to the differences between LoRa and NB-IoT in terms of access mechanisms and signal modulation, their collision separation approaches also differ significantly. Specific methods will be introduced in the next section.

### 2.3. LoRa Signal Seperation and Demodulation

To mitigate the reduction in system capacity caused by LoRa signal collisions, several studies have proposed MAC-layer or PHY-layer countermeasures within terrestrial LoRaWAN networks. About MAC-layer measures, Leonardi et al. [2] introduced lightweight modifications to the LoRaWAN medium access control (MAC) protocol, which aim to enhance the performance indicators of LoRaWAN uplink communications. Xu et al. [13] proposed an adaptive tuning mechanism for LoRaWAN networks, where devices adjust their transmission configurations to reduce packet collisions.

More studies focus on the PHY-layer measures. In this paper, we categorize PHY-layer LoRa collision separation methods into two representative groups:

1. Capture effect-based approaches, which rely on power disparities between overlapping signals;
2. FFT peak shape-based approaches, which exploit the spectral characteristics of LoRa chirps to identify multiple overlapping symbols.

The LoRa capture effect denotes the phenomenon in which the receiver can successfully decode the strongest signal even when multiple LoRa transmissions collide on the same channel and SF. Such successful decoding occurs only when the power of the strongest signal exceeds that of the interfering ones by a sufficient margin, typically around 6–10 dB [14]. Beyond power disparity, recent experimental studies have shown that LoRa capture performance is also strongly affected by timing alignment, residual CFO, and synchronization accuracy. For example, Lavric et al. [15] analyzed the capture effect in LoRa communications by examining packet collisions occurring on the same SF. The results indicate that successful demodulation under collision is mainly driven by the received signal strength, showing that packets with higher SNR or SIR ratios are more likely to be decoded. According to [16], practical impairments such as time misalignment and Doppler-induced frequency shifts reduce the effective orthogonality among LoRa signals using different spreading factors, thereby amplifying the impact of the capture effect in inter-SF collisions. However, for the same-SF signals, these impairments do not have a significant effect, as co-SF transmissions are inherently non-orthogonal.

To better exploit the capture effect, many studies employ successive interference cancellation (SIC) to separate colliding LoRa signals. SIC is a widely used technique for collision resolution in communication systems. Its fundamental principle is straightforward: the stronger signal is decoded and cancelled first, followed by the weaker one. Sant’Ana et al. [17] combined LoRa with SIC, exploiting the capture effect to demodulate the stronger signal and then cancelling its interference to recover the weaker one. Tesfay et al. [18] showed that a power domain SIC receiver can simultaneously demodulate multiple signals in an asynchronous uplink LoRa network. Ref. [19] introduced a precise drift-tracking module to mitigate timing and frequency offsets among superposed LoRa signals, greatly improving SIC performance. Moreover, these studies indicate that the attenuation of FFT peaks caused by synchronization deviations can also affect capture. In particular, when the received signal powers are comparable, a weaker signal with smaller synchronization deviations may be preferentially selected as the first signal for demodulation by the capture effect.

After dechirping a LoRa symbol, its FFT output exhibits a Dirichlet kernel-shaped peak [20]. Under perfect synchronization, the spectrum forms a single, symmetric peak at fixed FFT bins. Meanwhile, the FFT peaks of interfering signals often become asymmetric or distorted. Leveraging these peak-shape variations, numerous studies have developed methods for separating colliding LoRa signals. CoLoRa [21] builds by further exploiting the FFT energy leakage patterns induced by symbol-level arrival time offsets. By combining these features with interleaved FFT windowing and an iterative peak recovery mechanism, it enables the separation of multiple colliding LoRa signals without requiring any power disparity. However, when the colliding signals exhibit nearly perfectly aligned symbol timings, effective separation becomes infeasible. TnB [22] introduces a multi-feature joint-matching algorithm. It simultaneously exploits symbol boundaries, carrier frequency offset (CFO), and peak height history to achieve more robust discrimination of FFT peaks originating from different terminals. CoRa [23] proposes a collision-resistant LoRa symbol detector with low computational complexity. Instead of relying on conventional peak detection, CoRa employs a Bayesian classifier that operates on features extracted directly from the dechirped symbol waveform. However, CoRa is primarily designed for symbol-wise detection and does not explicitly exploit inter-symbol dependencies or joint demodulation across consecutive symbols. Moreover, although CoRa relaxes the reliance on peak detection, it still assumes reasonably accurate synchronization and residual offset compensation provided by the underlying receiver. Above all, this kind of approach requires highly accurate time-frequency synchronization, and the synchronization results for overlapping signals are often far from ideal.

Meanwhile, several studies have begun to apply machine learning to LoRa signal demodulation. By replacing traditional mathematical models with neural networks, these approaches are no longer constrained by specific signal features. Instead, they extract abstract information related to separation and demodulation in a more direct manner and make use of it accordingly. C. Li [24] proposes a neural-enhanced demodulation framework called NELoRa. By leveraging deep learning to extract multidimensional features of CSS signals that are overlooked by traditional methods, NELoRa enables reliable LoRa demodulation even under ultra-low SNR conditions. DeepLoRa [25] provides a CNN-based signal separation module whose input format more closely resembles the data structure used in traditional receivers. DeepLoRa demonstrates the feasibility of machine learning approaches for LoRa signal demodulation. However, DeepLoRa was not originally designed for signal separation. Therefore, during its training process, the dataset did not include samples involving signal collisions or more complex scenarios. In contrast, ConvLoRa considers multiple collision scenarios when constructing the dataset. Moreover, DeepLoRa cannot perform signal separation and can only demodulate based on the current symbol, whereas ConvLoRa can achieve signal separation by using reference chirps. In addition, DeepLoRa adopts a convolutional plus fully connected architecture rather than the fully convolutional architecture used in this work. As a result, DeepLoRa requires training a separate network for each SF, while the proposed ConvLoRa can operate with a single network.

To reduce the reliance of conventional demodulation methods on power disparity and precise synchronization, this paper proposes ConvLoRa. Unlike traditional SIC-based approaches, the proposed method does not rely on a power difference between colliding signals for successful separation. Although ConvLoRa also exploits the spectral characteristics of FFT peaks, the relevant features are extracted automatically by a neural network rather than relying on manually designed single-feature criteria. Furthermore, in contrast to methods such as CoLoRa and CoRa, which strongly depend on accurate time-frequency synchronization, ConvLoRa improves robustness to synchronization deviations through data augmentation during the training process.

## 3. System Model

### 3.1. LEO-IoT Channel Model

The large-scale path loss term ψ(l) captures the deterministic distance-dependent attenuation of the terminal–satellite link under a free-space propagation assumption. It represents the average fraction of transmitted power that is collected at the receiver after spherical spreading over the separation distance *l*. Accordingly, the free space large-scale attenuation can be expressed as

(1) $$\psi \left(l\right)={\left(\frac{\lambda }{4\pi l}\right)}^{2},$$

where λ denotes the carrier wavelength, *l* is the terminal–satellite distance, and ψ(l) is the corresponding large-scale power gain as a function of *l*.

In this paper, the small-scale fading of the terminal–satellite link is modeled using a shadowed Rician distribution, which jointly captures a dominant LoS component subject to slow shadowing and a diffuse multipath component. Let |h| denote the fading amplitude. The probability density function of |h| is given by

(2) $$f_{\left|h\right|}\left(\rho \right)={\left(\frac{2b_{0}m}{2b_{0}m+\Omega }\right)}^{m}\frac{\rho }{b_{0}}\exp\;\left(-\frac{\rho^{2}}{2b_{0}}\right)F\;\left(m,1,\frac{\Omega \rho^{2}}{2b_{0}\left(2b_{0}m+\Omega \right)}\right),$$

where ρ≥0 is the fading amplitude, F(·) denotes the confluent hypergeometric function F11(·), $2b_{0}$ is the average power of the diffuse multipath component, Ω is the average power of the (shadowed) LoS component, and *m* is the Nakagami-*m* shaping parameter governing the severity of the LoS shadowing. Moreover, the parameters $b_{0}\left(\theta \right)$, Ω(θ), and m(θ) depend on the elevation angle θ between the terminal and the satellite and are chosen according to the empirical expressions reported in [26].

The receive antenna gain of the LEO satellite is denoted by *G* and is modeled as a function of the off-axis angle with respect to the antenna boresight. Specifically, the gain pattern is characterized by an aperture-based formulation that accounts for the main lobe roll-off through Bessel functions of the first kind. The corresponding normalized angular variable and receive gain are expressed as

(3) $$\zeta =2.07123\frac{\sin(\phi )}{\sin(\phi_{3\mathrm{dB}})},$$

(4) $$G=\xi {\left(\frac{\pi R}{\lambda }\right)}^{2}{\left[\frac{J_{1}\left(\zeta \right)}{2\zeta }+36\frac{J_{3}\left(\zeta \right)}{\zeta^{3}}\right]}^{2},$$

where ξ is the antenna efficiency, *R* is the radius of the satellite receive antenna aperture, λ is the carrier wavelength, $J_{1}\left(\cdot \right)$ and $J_{3}\left(\cdot \right)$ denote the first- and third-order Bessel functions of the first kind, respectively, ϕ is the angle between the incident signal direction and the receive antenna boresight, $\phi_{3\mathrm{dB}}$ is the half-power (3 dB) beamwidth, and ζ is an auxiliary normalized angular variable defined for compactness, as in (4) [27].

By combining (1), (2), and (4), the relationship between the receive power and the transmit power $P^{r}$ can be expressed as follows:

(5) $$P^{r}=P^{t}G\psi \left(l\right){\left|h\right|}^{2}.$$

To clearly distinguish the constituent signals in the collision and their associated terminal/link parameters, we introduce the index γ∈{1, 2} to label the γ-th received component. Without loss of generality, the indices are ordered according to the signal arrival time at the satellite, i.e., a smaller γ corresponds to an earlier arrival at the receive antenna. With this notation, (5) can be written in a per-signal form as

(6) $$P_{\gamma }^{r}=P_{\gamma }^{t}G_{\gamma }\psi \left(l_{\gamma }\right){\left|h_{\gamma }\right|}^{2},$$

where $P_{\gamma }^{r}$ and $P_{\gamma }^{t}$ denote the received and transmitted powers of the γ-th terminal, respectively, $G_{\gamma }$ is the satellite receive antenna gain in the direction of the γ-th terminal, $\psi (l_{\gamma })$ is the large-scale free space power gain as a function of the corresponding terminal–satellite distance $l_{\gamma }$, and $|h_{\gamma }{|}^{2}$ is the small-scale fading power gain for the γ-th link.

According to the received power of the γ-th signal component, denoted by $P_{\gamma }^{r}$, the corresponding signal-to-noise ratio (SNR) is defined as

(7) $${\mathrm{SNR}}_{\gamma }=10\left|\log_{10}\left(\frac{P_{\gamma }^{r}}{P^{n}}\right)\right|\left(\mathrm{dB}\right),$$

where $P^{n}$ denotes the noise power.

Meanwhile, power disparity is an important parameter that affects the demodulation performance of the separation algorithm. We denoted it as PR (Power Ratio), which is defined as

(8) $${\mathrm{PR}}_{\gamma_{1},\gamma_{2}}=10\left|\log_{10}\left(\frac{P_{\gamma_{1}}^{r}}{P_{\gamma_{2}}^{r}}\right)\right|\left(\mathrm{dB}\right).$$

**Figure 1:**
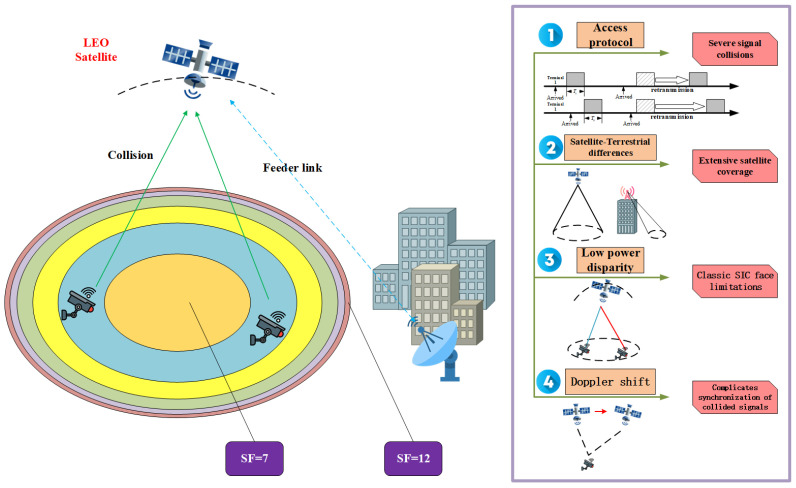
Illustration of the basic operating scenario for LEO-LoRaWAN.

In addition to the intrinsic characteristics of satellite channels, the SNR and the PR of colliding signals are also affected by the SF allocation scheme. Moreover, the SF allocation strategy influences the collision occurrence rate. A proper SF allocation scheme can reduce the on-air time of nearby terminals while ensuring the link quality of distant terminals. An SF allocation scheme that balances the collision power among terminals using different SFs was proposed in [28], and the corresponding beam partitioning is illustrated in Figure 1. In the SF partitioning, the smallest region is a circular area centered at the sub-satellite point, while the other SF regions are annular. Smaller SF values correspond to larger areas and include more terminals. The specific partition ratios can be found in [28]. Under this partitioning scheme, the collision probability in each SF region is essentially equal. Based on the above SF allocation method and the LEO channel conditions, numerical simulations are conducted to evaluate the PR between colliding signals.

To evaluate and quantify signal collisions in LEO-IoT scenarios, particularly the PR distribution among colliding signals, we conduct simulation experiments using Python and STK. The orbital altitude of the LEO satellite is 550 km, and the half-beamwidth is $\phi_{3dB}=43.75{}^{\circ}$. The geographical positions of terminals within the beam coverage are uniformly distributed over the spherical cap. The transmission times of terminals inside the beam coverage follow a Poisson distribution, and each transmission randomly carries 50–100 bytes of data. Finally, the distribution of the signal power ratios is obtained as Table 1. Terminal Percent denotes the proportion of signals with the corresponding SF among all collided signals.

From the table, it can be observed that in most cases the PR at the satellite does not satisfy the power disparity required by the capture effect. Consequently, conventional SIC algorithms may become ineffective for LoRa signal separation in LEO-IoT scenarios.

### 3.2. LoRa Signal

**Figure 2:**
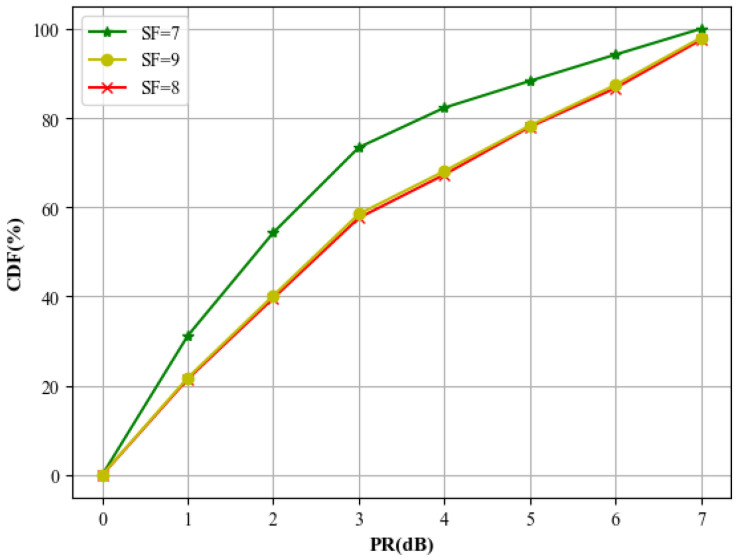
CDF curves for different SFs (corresponding to Table 1).

Figure 2 illustrates the basic PHY-layer frame structure of a LoRa signal. The LoRa frame consists of two main parts: the preamble and the PHY payload. The preamble is employed for signal detection and synchronization during the demodulation process, whereas the PHY payload is used for information transmission. The preamble comprises three components: the variable preamble (VP), the sync word (SW), and the start frame delimiter (SFD). Among these components, the VP typically contains eight standard up-chirp symbols and is primarily responsible for signal detection, frequency synchronization, and sampling-time alignment. The SW enables rapid identification of different LoRa networks and is generally formed by two identical modulated LoRa symbols. The SFD is composed of a 2.25-symbol down-chirp and is used together with the VP to achieve fine time-frequency synchronization.

LoRa signals, as a specific implementation of chirp spread spectrum (CSS) modulation, exhibit a linearly time-varying instantaneous frequency. The transmitted signal with an initial phase of 0 degrees at an IoT terminal can be expressed as

(9) $$s\left(t,x\right)=\left\{\begin{array}{cc} e^{j\pi \left(\frac{B}{M}t+2\frac{x}{M}B\right)t},\; & 0\leq t<T(x)\\ e^{j\pi \left(\frac{B}{M}t+2f\frac{x}{M}B-2B\right)t},\; & T\left(x\right)\leq t<T_{s} \end{array}\right.,x\in \left\{0,1,\ldots ,M-1\right\},$$

where *x* denotes the modulation value of the LoRa symbol, *t* is the continuous-time index within one symbol interval, $T_{s}$ is the LoRa symbol duration, $M=2^{\mathrm{SF}}$ is the symbol length, and *B* is the signal bandwidth. T(x) denotes the frequency hopping duration:

(10) $$T\left(x\right)=\frac{M-x}{B}.$$

**Figure 3:**
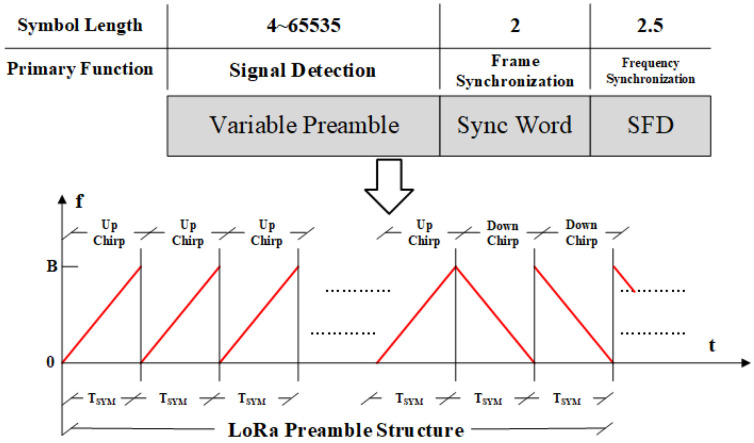
The variable preamble comprises eight up-chirps, and the start frame delimiter comprises 2.25 down-chirps.

According to the frame structure shown in Figure 3 and the expression in (9), the time-domain representation of the signal y(t,X) can be expressed as

(11) $$y\left(t,X\right)=\left\{\begin{array}{c} s\left(t\;\mathrm{mod}\;T_{s},0\right)\;0\leq t<8T_{s},\\ s\left(t\;\mathrm{mod}\;T_{s},X^{\mathrm{SW}}\left[\left\lfloor t/T_{s}-8\right\rfloor \right]\right)\;8T_{s}\leq t<10T_{s},\\ \bar{s(t\;\mathrm{mod}\;T_{s},0)}\;10T_{s}\leq t<T^{p},\\ s\left(\left(t-T^{p}\right)\;\mathrm{mod}\;T_{s},X\left[\left\lfloor t/T_{s}-12.25\right\rfloor \right]\right)\;T^{p}\leq t<\left(T^{p}+N^{s}T_{s}\right), \end{array}\right.$$

where $N^{s}$ denotes the number of payload symbols, $T^{p}$ denotes the preamble duration, $X^{\mathrm{SW}}$ represents the vector of modulation values of the SW, and X represents the vector of modulation values of the payload:

(12) $$X=\{x^{1},x^{2},\ldots ,x^{N^{s}}\}.$$

The composite baseband signal received at the satellite is denoted by z(t) over a total observation interval of duration *T*. It consists of Γ simultaneously collided uplink transmissions, where the γ-th component is associated with the γ-th arriving terminal signal. Each received component is modeled as a delayed version of the terminal-generated waveform, scaled by the transmit power and impaired by a Doppler-induced frequency offset and an unknown initial carrier phase. Accordingly, the received signal can be written as

(13) $$\begin{array}{cc} z(t) & =\sum_{\gamma =1}^{\Gamma }\sqrt{P_{\gamma }^{t}}\;y_{\gamma }\left(t-\tau_{\gamma },X_{\gamma }\right)\;e^{j(2\pi \Delta f_{\gamma }^{d}t+\varphi_{\gamma })}+w\left(t\right),\\ \; & 0\leq \tau_{1}\leq \cdots \leq \tau_{\Gamma }<T. \end{array}$$

where Γ denotes the number of colliding signals, $P_{\gamma }^{t}$ is the transmit power of the γ-th terminal, $y_{\gamma }\left(t-\tau_{\gamma },X_{\gamma }\right)$ is the corresponding transmitted baseband waveform parameterized by the modulation value vector $X_{\gamma }$ and delayed by the arrival time $\tau_{\gamma }$ at the receiver, $\Delta f_{\gamma }^{d}$ is the Doppler-induced frequency offset of the γ-th signal, $\varphi_{\gamma }$ is its initial phase, w(t) denotes additive noise, and *T* is the total sampling duration.The received analog waveform is sampled at rate $F_{s}=B$ to obtain the discrete-time sequence z[n], which is used for all subsequent processing. In the subsequent experiments, the arrival time of each signal follows a uniform distribution within the duration of the preceding signal. The probability distribution of the arrival time can be expressed as

(14) $$\begin{array}{ccc} \; & p\left(\tau_{0}=t\right)=1/\left(T-T^{p}-N_{0}^{s}T_{s}\right) & 0\leq t<T-T^{p}-N^{s}T_{s},\\ \; & p\left(\tau_{i}=t\right)=1/\left(T-T^{p}-N_{i-1}^{s}T_{s}\right) & \tau_{i-1}\leq t<\tau_{i-1}+T^{p}+N_{i-1}^{s}T_{s}, \end{array}$$

Moreover, the transmitting terminals of all signals are uniformly distributed within each SF region.

## 4. Design of ConvLoRa

In this paper, an efficient collision separation method is proposed for collided LoRa signals in LEO-IoT scenarios. We design a demodulation network that employs a Fully Convolutional Network (FCN) Model to extract the time-frequency features of the received symbols and estimate their corresponding demodulated values. The proposed demodulation network is capable of accurately recovering symbols under low PR conditions and imperfect synchronization in the presence of interfering peaks. In a satellite receiver, the demodulator normally operates under a single-signal scenario, while the sampling module continuously records the time-domain signal. If the signal is successfully demodulated, the stored samples are discarded. Otherwise, once a collision is detected during the demodulation process, the conventional demodulation module is interrupted. After all incoming signals are fully captured, the recorded samples z[n] are forwarded to an intelligent collision demodulation module for separation and further processing.

LoRa signals appear in the time domain as sequences of linearly frequency-varying waveforms. After de-chirping, the FFT representation exhibits one or multiple peaks, showing clear one-dimensional sequential characteristics. Moreover, the shape of each symbol is independent, making the signals well suited for processing with CNNs. CNNs are capable of effectively extracting local structural features from such one-dimensional patterns and are robust to noise and slight distortions. Therefore, a CNN-based approach can better capture the underlying structure of collided LoRa signals and improve demodulation performance under challenging conditions such as low power ratios and synchronization errors.

### 4.1. LoRa Detection and Synchronization

The LoRa detection method primarily follows the approach in [29] to estimate the number of collided signals, denoted by Γ, and the approximate start position of each individual signal. The number of signals is detected using the eight repeated up-chirps in the VP. After de-chirping and applying the FFT, these eight up-chirps produce eight identical FFT peaks. When such eight repeated peaks are observed, it indicates that a signal is present at that position, and the value of Γ can then be determined accordingly. In practical LEO-IoT scenarios, signals with different SFs are typically transmitted on different channels. Therefore, the specific SF value can be identified when the signal is received.

For ConvLoRa, fine-grained synchronization accuracy is not required for correct demodulation, but coarse synchronization results are still used. The synchronization method adopted in this paper is consistent with that used in conventional LoRa signal synchronization. Specifically, the procedure consists of three steps. First, the fractional carrier frequency offset (CFO) is estimated. Next, the fractional sampling time offset (STO) is estimated. Finally, the integer CFO and STO are estimated. For the detailed synchronization algorithm and processing flow, the reader is referred to [10]. After synchronization, the estimated starting sample index of the γ-th signal, ${\hat{\tau }}_{\gamma }$, and its Doppler frequency offset, ${\hat{f}}_{\gamma }^{d}$, can be obtained. Let ${\hat{n}}_{\gamma }^{0}$ be the estimated starting sample index of the γ-th LoRa signal in z[n], and let ${\hat{\epsilon }}_{\gamma }^{t}$ be the associated estimated fractional sampling time offset (STO). They satisfy

(15) $$\begin{array}{cc} \; & {\hat{n}}_{\gamma }^{0}=\left\lfloor {\hat{\tau }}_{\gamma }F_{s}\right\rceil ,\\ \; & {\hat{\epsilon }}_{\gamma }^{t}={\hat{\tau }}_{\gamma }F_{s}-{\hat{n}}_{\gamma }^{0}, \end{array}$$

where ⌊⌉ denotes rounding to the nearest integer. Similarly, under the condition $F_{s}=B$, the fractional CFO factor is given by

(16) $${\hat{\epsilon }}_{\gamma }^{f}=M{\hat{f}}_{\gamma }^{d}/B-\left\lfloor M{\hat{f}}_{\gamma }^{d}/B\right\rceil .$$

In this work, we classify the synchronization stage into three regimes: fully synchronized, partially synchronized, and weakly synchronized.

In conventional LoRa reception and most LoRa separation methods, the signal needs to be fully synchronized. The following conditions should be satisfied:

(17) $$\begin{array}{cc} \; & n_{\gamma }^{0}={\hat{n}}_{\gamma }^{0},\\ \; & \left\lfloor Mf_{\gamma }^{d}/B\right\rceil =\left\lfloor M{\hat{f}}_{\gamma }^{d}/B\right\rceil ,\\ \; & \left|\left(\epsilon_{\gamma }^{f}-{\hat{\epsilon }}_{\gamma }^{f}\right)-\left(\epsilon_{\gamma }^{t}-{\hat{\epsilon }}_{\gamma }^{t}\right)\right|<\frac{1}{2}, \end{array}$$

where the integer components of the STO and the CFO have been fully removed. Moreover, the residual fractional STO and CFO induce an FFT-bin displacement that remains within the current bin (i.e., it does not cross the adjacent FFT-bin boundary).

To reduce the strong dependence of collision-signal demodulation algorithms on precise synchronization results, we select an appropriate up-chirp in the preamble’s VP as a reference chirp and form a matrix with the time-domain samples of the subsequent payload symbols. This approach enables quite accurate demodulation, even when the signals are partially synchronized. The synchronization requirements of the proposed signal separation algorithm are summarized as follows:

(18) $$n_{\gamma }^{0}={\hat{n}}_{\gamma }^{0}.$$

Moreover, via data augmentation, we enable ConvLoRa to perform demodulation under further synchronization deviations; i.e., in the weakly synchronized regime. The conditions for weakly synchronized are given as follows:

(19) $$\left|n_{\gamma }^{0}-{\hat{n}}_{\gamma }^{0}\right|\leq M/4.$$

### 4.2. FFT-CNN

To more effectively extract time-frequency features from chirp signals for reliable demodulation, this work designs a fully convolutional module, referred to as FFT-CNN. The FFT-CNN constitutes a core component of the demodulation network. The architecture is inspired by SegNet [30], and is capable of transforming LoRa signals of different scales into a unified-scale set of feature vectors required for signal separation. The main characteristic of SegNet is that it stores the indices from max-pooling during the encoding stage and uses these indices for non-learned upsampling during the decoding stage, thereby restoring spatial structure while reducing parameters and memory usage. In this work, this architecture is adapted from a two-dimensional network designed for image processing to a one-dimensional convolutional network that is more suitable for signal processing. Moreover, time-domain and frequency-domain branches are used to extract features simultaneously, enabling LoRa signals of different scales to be transformed into a unified set of feature vectors required for signal separation.

**Figure 4:**
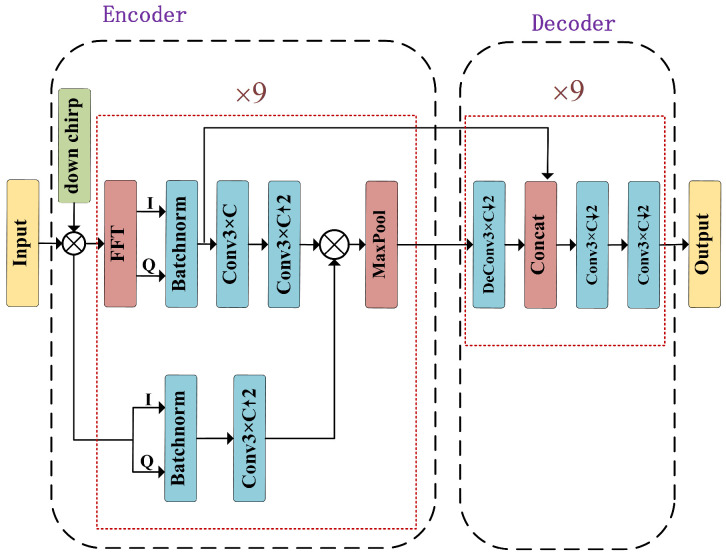
The FFT-CNN serves as one of the fundamental building blocks of the demodulation network.

The FFT-CNN is divided into two parts: an encoder and a decoder. In the encoder module, the time-domain and frequency-domain information are processed in two parallel streams, and the channel dimension of the feature maps is progressively increased. Feature fusion is then performed by element-wise multiplication of the resulting feature maps. The decoder module successively reduces the channel dimension to extract the demodulation-related representations as shown in Figure 4. All convolutional layers in the forward pass of the FFT-CNN are fully convolutional, ensuring that the input and output signal lengths remain identical; this enables robust feature extraction under different SFs. The FFT-CNN takes two input channels corresponding to the I/Q components and preserves the channel dimensionality at the output. To align with the FFT-spectrum characteristics of dechirped LoRa signals, all convolutional-layer padding is implemented using circular padding. Given that LoRa signals are typically received at low SNRs, dropout layers and other explicit regularization techniques are not employed in the FFT-CNN. Let $\mu^{\mathrm{FFT}}\left(\cdot \right)$ denote the inference function of the FFT-CNN.

### 4.3. Demodulation Model

#### 4.3.1. Demodulation Model Structure

The input to the demodulation network comprises the reference preamble and the symbols to be demodulated. The demodulation model is denoted by μ and takes two inputs: the reference chirp h extracted from the VP of the LoRa signal, and the symbol set R to be demodulated. The set R consists of the time-domain samples of one or more symbols from the payload of the same signal corresponding to h. We extract a reference chirp from the VP to provide the demodulation network with a reliable waveform anchor. For a given signal, the timing offset and carrier-frequency offset impose nearly identical distortions on both the reference chirp and the subsequent payload symbols. Moreover, their dechirped FFT spectra exhibit comparable peak magnitudes, which facilitates the demodulation model identifying the peak corresponding to the desired signal in the presence of collisions. With this reference information, the proposed demodulation model can still operate properly even when residual synchronization deviations exist.

The demodulation model consists of two FFT-CNN modules. One FFT-CNN is applied to the reference chirp h, while the other processes the time-domain symbol samples contained in R. Feature fusion is performed by element-wise multiplication of the feature maps generated by the two FFT-CNN modules. The fused features are further processed by a CNN module that reduces the two-channel feature maps to a single channel, yielding the output O (⊙ denotes element-wise multiplication). This process can be expressed as

(20) $$O=\mu^{\mathrm{CNN}}\left(\mu_{1}^{\mathrm{FFT}-\mathrm{CNN}}\left(h\right)⊙\mu_{2}^{\mathrm{FFT}-\mathrm{CNN}}\left(R\left[i\right]\right)\right),$$

where $\mu^{\mathrm{CNN}}$ denotes the final convolutional module used to produce the output, and $\mu_{1}^{\mathrm{FFT}-\mathrm{CNN}}$ and $\mu_{2}^{\mathrm{FFT}-\mathrm{CNN}}$ denote two internal FFT-CNN blocks with different learnable parameters for processing h and R, respectively.

The output O generated by demodulation model μ is a real-valued matrix, whose row dimension corresponds to the number of input symbols, and whose column dimension is determined by the SF and matches the symbol length, which is expressed as

(21) O=μ(h,R).

The demodulation result of the proposed network is derived from its output vector O and can be expressed as

(22) $$\begin{array}{cc} P_{\gamma } & =\mathrm{softmax}(\mu \left(h_{\gamma },R_{\gamma }\right)),\\ {\hat{X}}_{\gamma } & =\arg\max_{j\in \{0,\ldots ,M-1\}}\mu \left(h_{\gamma },R_{\gamma }\right)\left[j\right], \end{array}$$

where ${\hat{X}}_{\gamma }$ denotes the estimated value of the symbols of the γ-th signal, and $P_{\gamma }$ represents the corresponding a posteriori probability distribution over all candidate symbol values produced by the demodulation model.

#### 4.3.2. Demodulation Model Training Procedure

The first step in the training process is the generation of training data. All training data are generated by using SciPy and PyTorch in Python, in conjunction with the satellite simulation software STK. The detailed channel parameter settings and the LoRa signal format are consistent with those described in the Section 3. A total of 1000 collision signal samples are generated through simulation, which are randomly divided into train and test sets with a ratio of 7:3. The test set is not used for subsequent Monte Carlo experiments; instead, it is used to observe the training performance of the model. Although 1000 collision samples is not a large number, each sample contains 2–3 signals and each signal includes 50–500 symbols. In addition, data augmentation is used as a supplement.

The model takes as input the time-domain vector of a length-*M* payload segment from the collision signal samples. The training set contains 69,530 symbols, while the test set contains 30,142 symbols.

In the training set, each original received signal z[t] contains all collided signals. Before synchronization starts, the SF of the collided signals is assumed to be known. From the synchronization procedure, we obtain the estimated number of collided signals Γ, the estimated starting sample index of each signal in z[n], denoted by ${\hat{n}}_{\gamma }^{0}$, and the estimated Doppler frequency offset ${\hat{f}}_{\gamma }^{d}$. Moreover, all signals are accurately synchronized, where the synchronization conditions follow (18). Most signals in the training stage satisfy the partially synchronized condition.

In the training stage, the reference chirp h is obtained by randomly selecting one up-chirp from the eight up-chirps contained in the VP after signal synchronization. The set R consists of the time-domain samples of all symbols after synchronization. Based on this representation, multiple collided signals with the same SF are combined into a single batch for training. From a machine learning perspective, LoRa symbol demodulation can be formulated as a classification task. Accordingly, the cross-entropy loss is denoted as $L_{\mathrm{CE}}$, and is adopted as the training objective. Therefore, the output probability vector p is not indexed by the signal index γ. The batch size is denoted by $N^{b}$. $L_{\mathrm{CE}}$ is expressed as

(23) $$L_{\mathrm{CE}}=-\frac{1}{N^{b}}\sum_{i=1}^{N^{b}}x^{o}\left[i\right]\log P\left[i\right],$$

In the above equation, $x^{o}$ denotes the one-hot encoded representation corresponding to the ground-truth demodulated symbol.

To further enhance the robustness of ConvLoRa against synchronization imperfections, we perform data augmentation on the entire training set by injecting controlled random perturbations that emulate residual timing and frequency estimation errors. Concretely, for each training instance, we independently apply the following stochastic perturbations:

1. Timing jitter: with probability p=0.3, shift the estimated start index ${\hat{n}}_{\gamma }^{0}$ by one sample; i.e., ${\hat{n}}_{\gamma }^{0}\leftarrow {\hat{n}}_{\gamma }^{0}\pm 1$;
2. Sample dropout: with probability p=0.1, randomly set the corresponding time-domain sample to zero.

This augmentation procedure exposes the model to a spectrum of plausible synchronization deviations during training, thereby improving generalization and preventing severe performance degradation under mild synchronization deviations at inference time. During augmentation, we inject a large number of weakly synchronized signal samples to enhance the network’s adaptability.

Many existing collision separation methods assume that the number of symbols $N^{s}$ is known a priori, or equivalently, that the header carrying the symbol length information can be reliably decoded. However, such assumptions may not hold in LEO-IoT scenarios. To better address LoRa signal collision separation under variable packet lengths, this study adopts a multi-task learning (MTL) framework that jointly integrates symbol demodulation and symbol detection within the proposed Demodulation Network.

In this paper, we use the sum of its outputs O as the criterion to decide whether the current window corresponds to a preamble symbol, which is denoted as c:

(24) c[i]=∑O[i]

The objective of this part is to formulate a binary classification problem based on c to address the symbol detection task in LoRa collision scenarios. When c>0, the corresponding chirp is regarded as containing a valid symbol; otherwise, it is considered to contain no valid signal. To ensure that both the training and test sets are suitable for the proposed symbol-detection training, we further augment the dataset described above by a factor of two by constructing a balanced binary-classification set. Specifically, besides the length-*M* time-domain LoRa symbol segments used as positive instances, we randomly extract length-*M* windows from time regions that do not contain the currently demodulated signal and treat them as negative instances. The number of negative samples is set equal to that of the positive samples to avoid class imbalance. The binary cross-entropy (BCE) loss is then defined as

(25) $$L_{\mathrm{BCE}}=-\frac{1}{2N^{b}}\sum_{i=1}^{2N^{b}}\left[b\left[i\right]\log\sigma \left(c\left[i\right]\right)+\left(1-b[i]\right)\log\left(1-\sigma (c[i])\right)\right],$$

(26) $$\sigma \left(c\right)=\frac{1}{1+\exp(-c)},$$

where $N^{b}$ denotes the number of positive (or negative) samples. The label vector is $b\in {\left\{0,1\right\}}^{2N^{b}}$. In particular, b[i]=1 indicates that the *i*-th sample corresponds to a valid LoRa chirp (positive sample), while b[i]=0 indicates a negative sample. The term c[i] is the corresponding scalar logit output by the detector for the *i*-th sample. The function σ(·) denotes the sigmoid function. The loss $L_{\mathrm{BCE}}$ is averaged over all $2N^{b}$ training instances.

Finally, the overall loss $L_{d}$ is formulated as a weighted combination of the two losses defined above, which can be expressed as

(27) $$L_{\mathrm{total}}=L_{\mathrm{CE}}+\alpha L_{\mathrm{BCE}},$$

where α is set as 0.1 in training.

**Figure 5:**
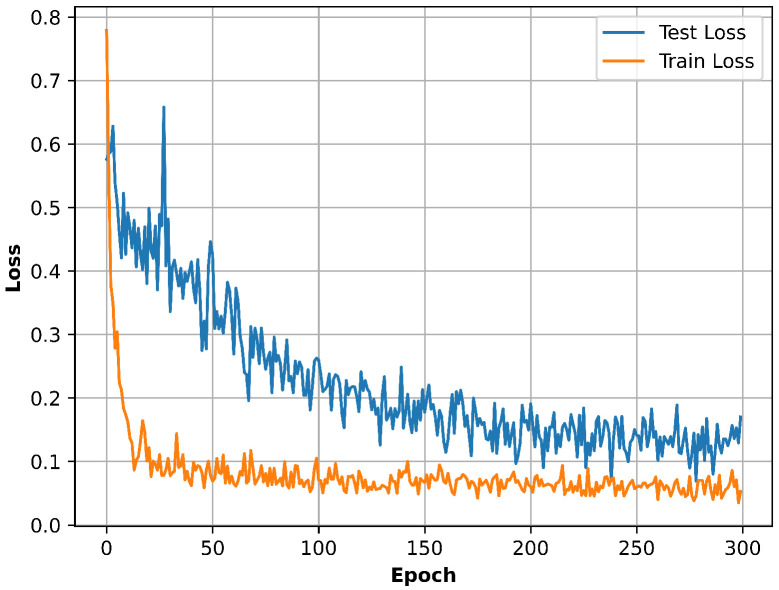
Training and test loss curves.

During training, the batch size $N^{b}$ is fixed to 256. The AdamW optimizer is employed for model optimization, together with a learning rate warm-up strategy. Specifically, the learning rate is initialized at $10^{-5}$ and linearly increased to $10^{-3}$ over the first 50 epochs. After the warm-up stage, if the loss $L_{\mathrm{total}}$ on the test set does not improve within 20 consecutive epochs, the learning rate is multiplied by a factor of 0.9. The learning rate is bounded below by $10^{-5}$. The evolution of the training loss is illustrated in Figure 5.

To determine suitable hyperparameters for the proposed CNN model, a grid search strategy was employed during the training phase. Specifically, several key hyperparameters, including the learning rate, batch size, and optimizer-related parameters, were systematically explored within predefined ranges. For each candidate combination of hyperparameters, the model was trained and evaluated on the validation dataset, and the configuration achieving the best validation performance was selected as the final setting.

The search ranges were defined according to commonly used values in deep learning practice. The learning rate was tested within the range of $10^{-4}$ to $10^{-2}$, while multiple batch sizes were considered to balance convergence stability and computational efficiency. Through this grid search process, the model hyperparameters were optimized to achieve improved training stability and overall performance.

### 4.4. Computational Complexity Analysis

This section analyzes the computational complexity of the proposed ConvLoRa demodulation scheme and compares it with conventional LoRa demodulation. Both the inference-stage complexity and the training-stage complexity are discussed in order to provide a comprehensive assessment.

#### 4.4.1. Baseline LoRa Demodulation Complexity

In conventional LoRa receivers, demodulation is performed by dechirping the received signal followed by an *M*-point FFT and peak detection, where $M=2^{\mathrm{SF}}$ denotes the number of samples per symbol. The dominant computational cost arises from the FFT operation, whose complexity scales as

(28) $$O\;\left(M\log_{2}M\right).$$

Additional operations such as point-wise complex multiplication for dechirping and peak searching contribute O(M) complexity and are therefore secondary. Consequently, the overall per-symbol complexity of conventional LoRa demodulation is FFT-dominated and increases logarithmically with the SF.

#### 4.4.2. Inference Complexity of the Proposed ConvLoRa Scheme

The proposed ConvLoRa demodulator replaces the FFT-based peak detection with a fully convolutional neural network architecture. Specifically, it consists of two parallel FFT-CNN branches for channel estimation and interference suppression, followed by a lightweight convolutional classifier for symbol decisions.

The inference complexity of ConvLoRa is dominated by convolution operations. For a one-dimensional convolution layer with input length *L*, kernel size *k*, input channels $C_{\mathrm{in}}$, and output channels $C_{\mathrm{out}}$, the number of multiply–accumulate operations (MACs) is given by

(29) $$\mathrm{MACs}=L\cdot C_{\mathrm{out}}\cdot \left(C_{\mathrm{in}}\cdot k\right).$$

Accordingly, the total inference complexity of ConvLoRa can be expressed as the sum of MACs over all convolutional layers in both FFT-CNN branches and the subsequent classifier. Since the proposed network is fully convolutional, the input length *L* is equal to $M=2^{\mathrm{SF}}$, enabling efficient adaptation to different SFs without retraining. The kernel size *k* is set to three in all layers. In the network, the number of output channels $C_{\mathrm{out}}$ in each layer is twice the number of input channels $C_{\mathrm{in}}$. Accordingly, the $C_{\mathrm{out}}$ in the preceding layer is equal to the $C_{\mathrm{in}}$ in the subsequent layer. The input channel number of the first layer is set to two, and the model consists of a total of nine layers.

#### 4.4.3. Training Complexity

The training complexity of ConvLoRa is significantly higher than its inference complexity and is primarily determined by the number of training samples, the network size, and the number of training epochs. Let *N* denote the number of training symbols and *E* the number of training epochs. The total training complexity can be approximated as

(30) $$O\;\left(E\cdot N\cdot C_{\mathrm{fb}}\right),$$

where $C_{\mathrm{fb}}$ represents the computational cost of one forward–backward propagation pass, which is typically in the order of two to three times the inference complexity. The number of training symbols is equal to the batch size, which is set to 256. As shown in Figure 5, the number of training epochs *E* is set to 300.

It is important to note that the training phase is performed offline on dedicated computing platforms and does not impose any computational burden on satellite devices. Once trained, the model parameters are fixed and only the inference-stage complexity is incurred during deployment. Therefore, the high training cost constitutes a one-time offline expense and does not affect the runtime complexity of the receiver.

Compared with conventional FFT-based demodulation, the proposed ConvLoRa scheme incurs higher computational complexity during inference due to convolution operations. However, this increase enables substantially improved robustness against synchronization deviations and same-SF interference. Moreover, the inference process can be efficiently accelerated using modern embedded GPUs or dedicated neural processing units, while the training phase remains fully decoupled from practical system operation.

### 4.5. LoRa Symbol Demodulation Procedure

Given the sampled signal z[n] acquired by the satellite receiver, we assume that the number of colliding signals Γ and the spreading factor SF are known. The synchronization module then outputs an estimate of the start index ${\hat{n}}_{\gamma }^{0}$.

The time-domain representation of the VP for the γ-th signal is denoted by $H_{\gamma }$. The time-domain representation used for subsequent symbol processing is denoted by $R_{\gamma }$. Since the exact number of symbols is unknown, the samples ranging from the payload start index ${\hat{n}}_{\gamma }^{0}+12.25\;M$ to the end of z[n] are regarded as a temporary symbol set $R_{\gamma }^{*}$:

(31) $$\begin{array}{cc} \; & H_{\gamma }\left[i\right]=z\left[{\hat{n}}_{\gamma }^{0}+iM:{\hat{n}}_{\gamma }^{0}+\left(i+1\right)M\right]⊙\exp\left(-j2\pi {\hat{f}}_{\gamma }^{d}t\right),\\ \; & 0\leq i\leq 7,\;H_{\gamma }\in C^{8\times M},\\ \; & t=[0,1,\ldots ,M-1]/B, \end{array}$$

(32) $$\begin{array}{cc} \; & R_{\gamma }^{*}\left[i\right]=z\left[12.25M+{\hat{n}}_{\gamma }^{0}+iM:12.25M+{\hat{n}}_{\gamma }^{0}+\left(i+1\right)M\right]⊙\exp\left(-j2\pi {\hat{f}}_{\gamma }^{d}t\right),\\ \; & 0\leq i\leq \left\lfloor (N^{T}-{\hat{n}}_{\gamma }^{0})/M-1\right\rfloor ,\\ \; & t=[0,1,\ldots ,M-1]/B. \end{array}$$

where $N^{T}$ denotes the number of samples in z[n].

The demodulation procedure can be broadly divided into three stages:

**(s1)**  Select the reference chirp from the VP as the reference.
**(s2)**  Compute the total number ${\hat{N}}_{\gamma }^{s}$ of symbols contained in each signal.
**(s3)**  Demodulate the symbols with reference chirp (h) and time domain of symbols (R).

#### 4.5.1. Select Reference Chirp

In the presence of multi-packet collisions, synchronization is prone to deviations. To ensure reliable demodulation even in the presence of such deviations, an up-chirp extracted from the VP is used as a baseline in this paper. In particular, the impacts of timing offset and carrier-frequency offset on the FFT spectra of the up-chirp and the subsequent LoRa symbols are equivalent, which enables the neural network to learn and match the correlated features. Moreover, this strategy along with neural network can largely suppress the adverse effects introduced by integer frequency offset and fractional timing offset.

Since the VP consists of repeated up-chirp symbols with a known demodulation value, a length-*M* window extracted from the VP can be used as a time-domain LoRa symbol sample with a known label. Following this idea, we construct a labeled time-domain symbol sequence from the received samples and denote it by $R_{\gamma }^{\mathrm{VP}}$. Specifically, each $R_{\gamma }^{\mathrm{VP}}$ contains $N^{\mathrm{sample}}$ LoRa symbols that are randomly extracted from the VP region. Let $k_{\gamma }\left[i\right]$ denote the starting sample index of the *i*-th extracted symbol. The *i*-th VP symbol sample is then formed as

(33) $$\begin{array}{cc} R_{\gamma }^{\mathrm{VP}}\left[i\right] & =z\;\left[k_{\gamma }\left[i\right]:k_{\gamma }\left[i\right]+M-1\right],\\ k_{\gamma }\left[i\right] & ∼\mathrm{Unif}\;\left(\left\{{\hat{n}}_{\gamma }^{0},{\hat{n}}_{\gamma }^{0}+1,\ldots ,{\hat{n}}_{\gamma }^{0}+7M\right\}\right),\;0\leq i\leq N^{\mathrm{sample}}-1, \end{array}$$

where z[·] denotes the discrete-time received sequence, *M* is the LoRa symbol length in samples, ${\hat{n}}_{\gamma }^{0}$ is the estimated start index of the VP for the γ-th signal, $k_{\gamma }\left[i\right]$ is the random starting sample index of the *i*-th extracted symbol, and $N^{\mathrm{sample}}$ is the number of extracted VP symbols (typically set to 10).

Then, the trained demodulation network μ is applied to all eight up-chirps within the VP from both signals to demodulate $R_{\gamma }^{\mathrm{VP}}$. The suitability of each up-chirp as the reference chirp is evaluated based on the average a priori probability corresponding to the actual symbol of $R_{\gamma }^{\mathrm{VP}}$ in the network output. Consequently, the index of the reference chirp within the VP is obtained according to the following expression:

(34) $$k_{\gamma }^{\mathrm{VP}}=\arg\max_{i\in \{0,1,\ldots ,7\}}\sum_{j=0}^{N^{\mathrm{sample}}-1}\left(\mathrm{softmax}\left(\mu \left(H_{\gamma }\left[i\right],R_{\gamma }^{\mathrm{VP}}\left[j\right]\right)\right)\left[M-k_{\gamma }\left[i\right]\mathrm{mod}M\right]\right).$$

Let the $k_{\gamma }^{\mathrm{VP}}$-th up-chirp in VP be denoted as reference chirp (h) of the γ-th signal. $h_{\gamma }$ remains unchanged throughout the entire demodulation process.

#### 4.5.2. Payload Length Estimation

As implied by (25), we formulate a binary classification task based on the sum of the model outputs, enabling the proposed demodulation model to obtain both the payload length estimate and the demodulation result within a single forward pass. Observing that all LoRa payload symbols are necessarily contiguous in the time domain, we estimate the number of symbols by cumulatively summing c and then selecting the index corresponding to

(35) $$\begin{array}{cc} \; & {\hat{N}}_{\gamma }^{s}=\arg\max_{i\in \{0,1,\ldots ,r\}}\sum_{j=0}^{i}c_{\gamma }\left[j\right],\\ \; & N_{\gamma }^{*}=\left\lfloor \left(N^{T}-{\hat{n}}_{\gamma }^{0}-N^{p}\right)/M\right\rfloor , \end{array}$$

where $c_{\gamma }\left[j\right]$ denotes the detector logit associated with the candidate length-*M* window for the γ-th signal, $N_{\gamma }^{*}$ is the length of $R_{\gamma }^{*}$ and ${\hat{N}}_{\gamma }^{s}$ is the resulting estimate of the number of payload symbols for that signal. Accordingly, the symbol set for demodulation, denoted by $R_{\gamma }$, can be determined as the first $N_{\gamma }^{s}$ rows of $R_{\gamma }^{*}$. When the payload length is fixed, the estimated ${\hat{N}}_{\gamma }^{s}$ can be directly validated.

#### 4.5.3. Demodulation

**Figure 6:**
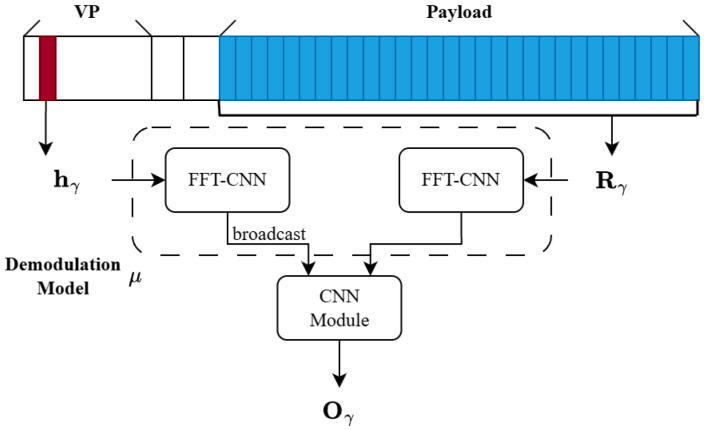
Simplified inference pipeline of the proposed ConvLoRa framework.

The overall demodulation process is illustrated in Figure 6. Given the reference preamble $h_{\gamma }$ obtained in the previous two steps and the payload symbol group $R_{\gamma }$ determined after payload length estimation, we follow the same inference procedure as in training: $h_{\gamma }$ and $R_{\gamma }$ are fed into the model, and a simple post-processing step yields an accurate demodulation result, as shown below:

(36) $${\hat{X}}_{\gamma }\left[i\right]=\arg\max_{j\in \{0,1,\ldots ,M-1\}}\mu \left(h_{\gamma }\left[i\right],R_{\gamma }\left[i\right]\right)\left[j\right]$$

However, in practical experiments, we observed that when an FFT spectral peak lies near the boundary between two adjacent bins, the demodulation model is more likely to be confused. To mitigate this issue, we additionally apply a frequency shift of 0.5 FFT bins to the received signal and perform demodulation on both the original and the shifted signals. The symbol posterior probability distributions obtained from the two demodulation runs are given as follows:

(37) $$\begin{array}{cc} \; & P_{\gamma }^{\prime }\left[i\right]=\mathrm{softmax}\;\left(\mu \;\left(h_{\gamma }⊙e^{-j2\pi \frac{B}{M}t},\;R_{\gamma }\left[i\right]⊙e^{-j2\pi \frac{B}{M}t}\right)\right),\\ \; & P_{\gamma }\left[i\right]=\mathrm{softmax}\;\left(\mu \;\left(h_{\gamma },\;R_{\gamma }\left[i\right]\right)\right),\\ \; & t=\left[0,1,\ldots ,M-1\right]/B. \end{array}$$

When selecting the estimated demodulation value, we use the larger confidence score between the two demodulation results as the criterion, which can be expressed as

(38) $${\hat{X}}_{\gamma }\left[i\right]=\arg\max_{j\in \{0,1,\ldots ,M-1\}}\;\max\left(P_{\gamma }\left[i,j\right],P_{\gamma }^{\prime }\left[i,j\right]\right),$$

where ${\hat{X}}_{\gamma }\left[i\right]$ denotes the final demodulated symbol value of the γ-th signal. This procedure is repeated until all Γ collided signals are successfully demodulated, after which the signal-separation process terminates. The pseudocode for the entire procedure is given as Algorithm 1.

**Algorithm 1:** ConvLoRa-Based LoRa Collision Demodulation

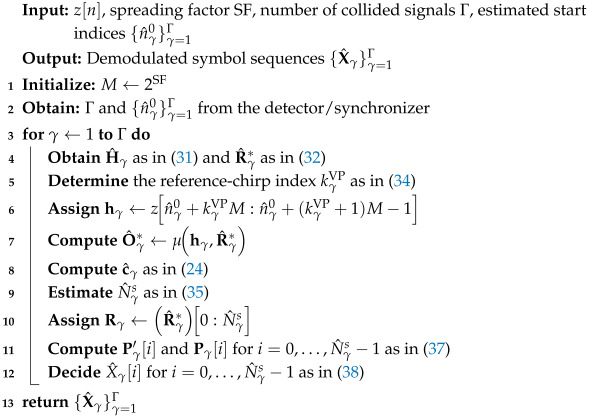



## 5. Results and Analysis

**Figure 7:**
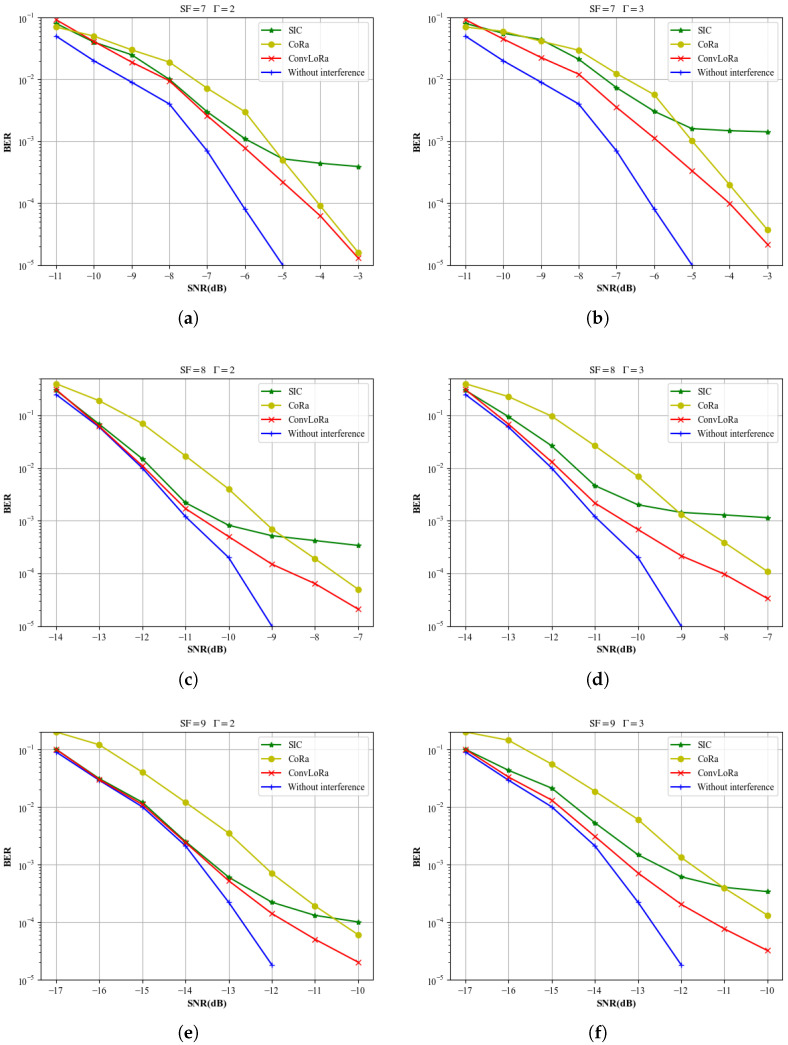
Comparison of demodulation BERs under different SFs and SNRs.

**Figure 8:**
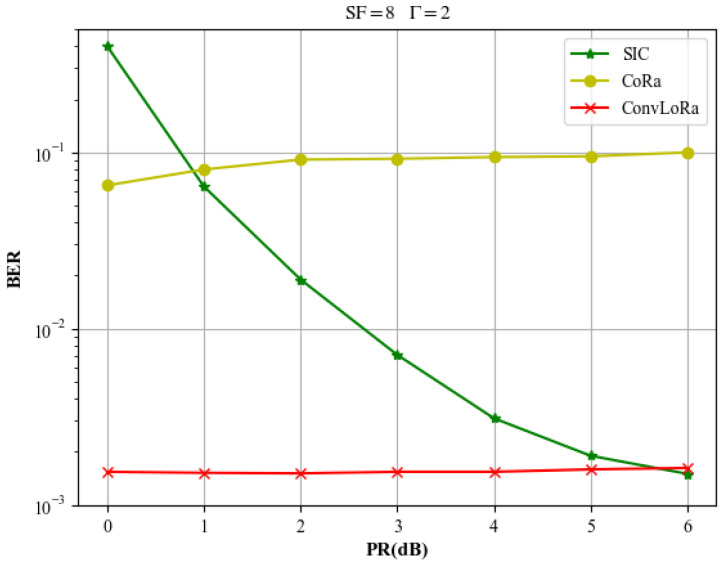
BER curves of different LoRa signal separation methods under various PRs.

**Figure 9:**
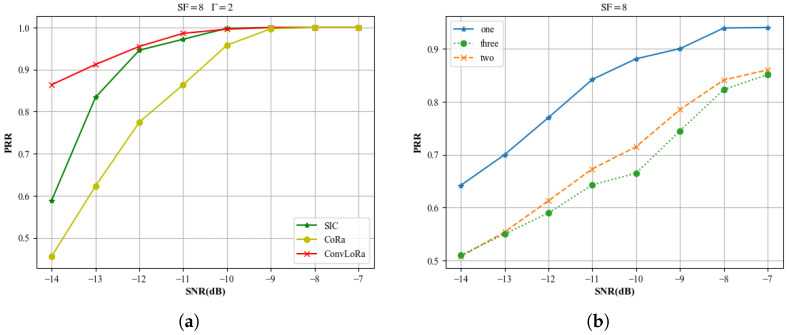
Different LoRa demodulation PRRs of various methods under different SNRs: (**a**) PRR versus SNR for different methods; (**b**) PRR versus SNR of ConvLoRa under different numbers of collisions.

**Figure 10:**
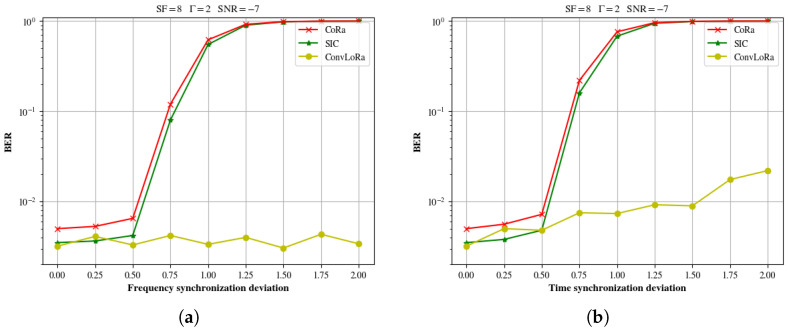
Performance variation of different algorithms under synchronization deviations: (**a**) BER curves of different methods under frequency offset; (**b**) BER curves of different methods under timing offset.

**Figure 11:**
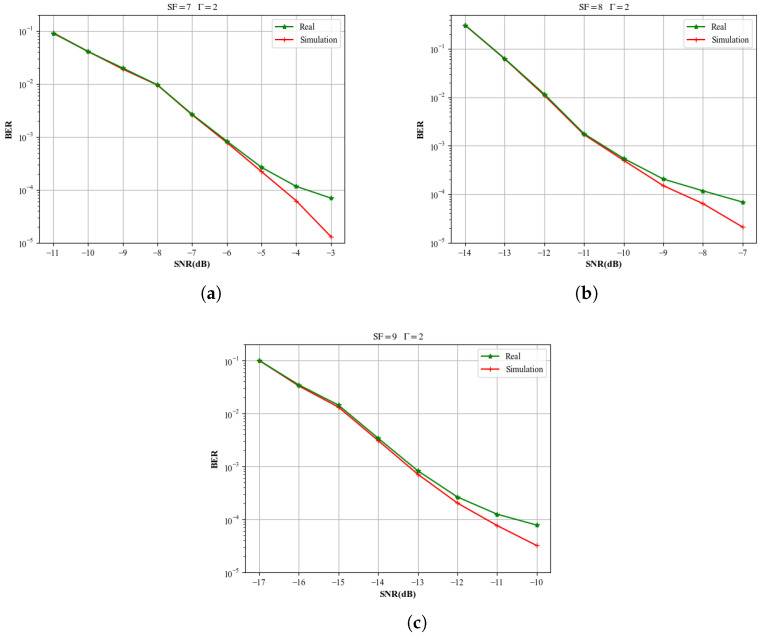
Performance discrepancy of the proposed method between real measurement data and Python simulation: (**a**) BER difference between simulation and experimental results at SF = 7; (**b**) BER difference between simulation and experimental results at SF = 8; (**c**) BER difference between simulation and experimental results at SF = 9.

This paper first evaluates the effectiveness of the proposed steps using simulated data, highlighting the improvements over conventional methods. For the *γ*th signal, the power ratios in this section’s experiments follow the distribution specified in Table 1 in the System Model. In the experiments shown in Figure 7, the power differences between the colliding signals follow the distribution described in Table 1. In Figure 8, to evaluate the separation performance under different PR conditions, the PR distribution among the signals does not follow that specified in Table 1. In the experiments shown in Figure 9, Figure 10 and Figure 11, the power differences between the colliding signals follow the distribution described in Table 1. During testing, every signal collides with another, with collision patterns fully randomized. All simulation experiments are conducted using the Monte Carlo method based on the LoRa signal format and the LEO channel. All evaluation samples are drawn from the test set described earlier. Moreover, for BER computation, we only consider signals that can be fully synchronized according to (17).

Figure 7 includes three scenarios with SF values of 7, 8 and 9. In the test samples used for Figure 7, all test data are successfully synchronized according to (17). The green curve represents the BER performance of the conventional SIC algorithm [19]. The yellow curve corresponds to CoRa, while the red curve denotes the proposed method in this paper. This article mainly compares the most advanced deep learning demodulation scheme DeepLoRa. Although CoLoRa and Nscale are efficient algorithms for handling conflicts, they usually perform better in scenarios with higher SNR or better synchronization. Considering the extremely low signal-to-noise ratio and large frequency offset of the LEO satellite link, we focus on the deep learning model that also has strong robustness. The blue curve shows the BER performance of the traditional demodulation method under the single-signal interference-free scenario. As can be observed from Figure 7, the proposed method achieves a more pronounced improvement in demodulation performance as the SNR decreases. Moreover, under more severe multi-packet collisions, it exhibits a smaller performance degradation compared with the baselines.

To facilitate a more in-depth performance analysis of different signal separation methods under different PRs, we consider a two-signal collision scenario with a spreading factor of SF=8 and a received signal-to-noise ratio of SNR=−12dB. Under this setting, extensive Monte Carlo simulations are conducted to evaluate the demodulation BER of different methods for varying PRs. Moreover, in the experiments, the PR value does not vary according to Table 1 but instead follows predefined values. The SNR of the signals is uniformly distributed between −14dB and −7dB. The corresponding results are illustrated in Figure 8. As can be observed from the figure, the proposed method significantly outperforms conventional SIC algorithms under different power ratio conditions, particularly in the low power ratio scenario. This characteristic makes the proposed approach well suited for LEO-IoT scenarios, where co-SF signals typically exhibit small power disparities.

In the dataset construction stage, we apply data augmentation by deliberately injecting samples with pronounced synchronization mismatches, thereby improving the model’s robustness against synchronization deviations. In the previous sections, we performed experiments under ideal synchronization conditions. To better demonstrate the robustness of the proposed algorithm under synchronization errors and to more closely reflect practical demodulation performance, the experiment shown in Figure 9 is conducted without applying any filtering or selection based on the signal synchronization condition. We employ the packet reception ratio (PRR) as an additional metric to evaluate the signal separation capability of different models. PRR is defined as the fraction of transmitted packets that are successfully received and correctly decoded at the receiver (typically the gateway) under a given experimental or simulation setting, which can be expressed as

(39) $$\mathrm{PRR}=\frac{N_{\mathrm{succ}}}{N_{\mathrm{tot}}}.$$

According to (39), a packet is counted in $N_{\mathrm{succ}}$ only when all payload symbols are correctly demodulated, regardless of whether the synchronization stage succeeds. This evaluation enables us to assess the capability of different separation methods to tolerate synchronization deviations under practical channel conditions. In the experiment shown in Figure 9b, all samples exhibit synchronization deviations, and the results demonstrate the separation performance of the proposed method under collisions involving different numbers of signals.

To further illustrate the performance of ConvLoRa in the presence of synchronization deviations, this paper evaluates the impact of carrier frequency offset and timing synchronization error under the conditions of SF=7 and SNR=−7dB. As shown in Figure 10a, the horizontal axis represents the magnitude of the frequency synchronization error, $|\epsilon_{\gamma }^{f}-{\hat{\epsilon }}_{\gamma }^{f}|$, while the vertical axis denotes the BER. The timing synchronization error is set to zero in this case. It can be observed that the proposed ConvLoRa algorithm exhibits strong robustness against frequency synchronization deviations. This robustness arises from the fact that, when a frequency offset is present, both the reference chirp and the received symbol experience identical frequency shifts in the time domain, which significantly reduces the probability of demodulation errors. As shown in Figure 10b, the horizontal axis corresponds to the magnitude of the timing synchronization error, $|\epsilon_{\gamma }^{t}-{\hat{\epsilon }}_{\gamma }^{t}|$, and the vertical axis represents the BER, while the frequency synchronization error is fixed to zero. Timing synchronization deviations have a pronounced impact on the performance of all considered methods; however, ConvLoRa remains the least affected. The underlying reason is consistent with the discussion above. In particular, timing offsets lead to incomplete symbol capture within the observation window, resulting in a reduction in the FFT peak magnitude and the introduction of interference from adjacent symbols. Conventional SIC demodulation schemes rely heavily on the absolute location of the FFT peak to recover the transmitted symbol, which substantially degrades their adaptability to synchronization deviations.

To further analyze the contribution of different branches, we conducted ablation experiments by removing either the time-domain or frequency-domain branch of the reference chirp and the target symbol in the network. The experiments were performed under Γ=2 and SNR=−9 in SF=8. We found that removing the time and frequency branches in the reference chirp increased the BER by 78% and 42%, respectively. Similarly, in the FFT-CNN used to process the symbol, removing the time and frequency branches increased the BER by 54% and 21%, respectively. It can be observed that the most critical component for ConvLoRa is the frequency branch of the FFT-CNN corresponding to the reference chirp.

In addition to the simulation tests, we employ the IZT C3040 satellite channel emulator to model an LEO satellite channel. Two AD9361 transceiver modules generate the LoRa baseband signal at a carrier frequency of 1.6GHz, and Gaussian white noise is produced by an R&S SMBV100A signal generator. Figure 11 demonstrates that the proposed separation method generalizes well to realistic communication scenarios. In the experimentally measured environment, two AD9361-based LoRa development boards were used to transmit LoRa signals in the same format as in the simulations. During the experiments, a random Doppler variation rate of 500–800 Hz/s is introduced. The signal-to-noise ratio was adjusted by varying the power of an external noise source. The figure compares the performance of the proposed intelligent LoRa separation algorithm under simulated and measured signal environments. When the SNR is below −11 dB, the performance gap between the simulated and measured environments is negligible; for SNRs above −11 dB, the measured environment exhibits a slight performance degradation relative to the simulated case. These findings validate that the proposed intelligent demodulation scheme can achieve reliable signal separation and demodulation under low power-difference conditions in satellite links.

Under the condition of a two-signal collision with SNR = −9 dB and SF = 8, the BER of the proposed method is 21% that of CoRa and 28% that of the SIC-based method. For LoRa signal collisions under different SFs, the performance difference between the data generated by the channel simulator and the simulated data is less than 10%.

Although ConvLoRa has many advantages, it also has two significant limitations. First, it requires considerable computational resources. As mentioned earlier, the complexity of ConvLoRa is relatively high, which imposes certain requirements on the satellite hardware. Second, extracting the reference chirp requires that the time difference between two signals be sufficiently large; specifically, it must be greater than half a symbol duration to successfully extract the reference chirp.

To accelerate inference and better match the deployment requirements of IoT terminals, we export the proposed model to ONNX for inference. Benefiting from the graph-level optimizations in ONNX Runtime, including node elimination, constant folding, and more advanced operator fusion and layout optimizations, the inference latency of the convolutional layers can be significantly reduced. Previous studies [31] have reported the deployment of NVIDIA Jetson TX2i GPU modules for on-orbit data processing in LEOP missions. As a representative example, Jetson TX2i has been demonstrated as a cost-effective and flight-proven GPU-based computing platform for LEO applications. The Jetson TX2i GPU is based on the same Pascal architecture as the NVIDIA GTX 1050 Ti and provides a comparable level of computational capability. Motivated by these prior in-orbit demonstrations, some additional inference experiments in this work are conducted on an NVIDIA GTX 1050 Ti to evaluate the feasibility and performance of the proposed approach. Experimental results show that the average inference latency per execution is below 200 ms. Furthermore, the achieved throughput exceeds 200 LoRa symbols per inference (for SF=8), enabling the parallel inference of three to four groups of colliding signals.

## 6. Conclusions

With the development of LEO satellite IoT technology, the number of terminals covered by LEO-IoT systems continues to increase, leading to more severe uplink packet collisions. The conventional SIC approach struggles to cope with the low-power disparity characteristic of LEO-IoT signals. Therefore, we propose a deep learning-based separation algorithm to enhance the capacity of LEO-IoT. Through both simulation tests and real-world data measurements, we demonstrate that, under the low-power disparity conditions typical of LEO-IoT, the proposed method significantly outperforms traditional SIC in separating LoRa signals with the same SFs, and exhibits improved robustness to synchronization deviations.

In the future, we will focus on designing a demodulation procedure capable of effectively handling time-varying Doppler shifts, addressing the interference caused by Doppler frequency offsets at higher SFs in LEO-IoT scenarios. Meanwhile, we plan to further simplify the proposed model and deploy it on FPGA-based platforms to accelerate inference and reduce the inference latency. We will improve the synchronization algorithms to handle more complex overlapping scenarios, particularly cases involving multiple signal overlaps and low-latency overlaps between colliding signals. Furthermore, in future work, we will improve the ConvLoRa architecture to enable it to adapt to LoRa signal demodulation under high SFs and highly dynamic Doppler conditions.

## Figures and Tables

**Table 1:** Power disparity statistics across different SF regions.

SF	PR<6dB	PR<3dB	PR<1dB	Terminal Percent
SF=7	94.2%	73.4%	31.2%	45.0%
SF=8	86.7%	57.6%	21.5%	25.7%
SF=9	87.4%	58.5%	21.8%	14.4%

## Data Availability

The data supporting the reported results are generated from simulations and are available from the corresponding author upon reasonable request.
